# Inductive role of the brown alga *Sargassum polycystum* on growth and biosynthesis of imperative metabolites and antioxidants of two crop plants

**DOI:** 10.3389/fpls.2023.1136325

**Published:** 2023-02-24

**Authors:** Soha Mohammed, Mostafa M. El-Sheekh, Saadia Hamed Aly, Maha Al-Harbi, Amr Elkelish, Aziza Nagah

**Affiliations:** ^1^ Botany and Microbiology Department, Faculty of Science, Banha University, Benha, Egypt; ^2^ Botany Department, Faculty of Science, Tanta University, Tanta, Egypt; ^3^ Department of Biology, College of Science, Princess Nourah bint Abdulrahman University, Riyadh, Saudi Arabia; ^4^ Biology Department, College of Science, Imam Mohammad ibn Saud Islamic University (IMSIU), Riyadh, Saudi Arabia; ^5^ Botany and Microbiology Department, Faculty of Science, Suez Canal University, Ismailia, Egypt

**Keywords:** biostimulation, seaweeds, polysaccharides, antioxidants, animal nutrition, soil health

## Abstract

The potential of macroalgae as biostimulants in agriculture was proved worthy. *Vicia faba* and *Helianthus annuus* are socioeconomic crops owing to their increasing demand worldwide. In this work, we investigated the energetic role of seed presoaking and irrigation by the brown seaweed, *Sargassum polycystum* aqueous extract (SAE) on certain germination and growth traits, photosynthetic pigments, carbohydrates, phenolics, flavonoids, and the total antioxidant activity. Compared to the control plants, our consequences revealed that seeds that received the SAE improved all the germination and growth criteria for both crop plants. Furthermore, the SAE significantly increased the carotenoids, total photosynthetic pigments, and total carbohydrates by (14%, 7%, and 41%) for *V. faba* and (17%, 17%, and 38%) for *H. annuus*, respectively. Phenolics and flavonoids were significantly induced in *Vicia* but slightly promoted in *Helianthu* plants, whereas the total antioxidant activity in both crops non significantly elevated. Even though The NPK contents were significantly stimulated by the SAE in *Vicia* plants, the effect was different in *Helianthus*, where only nitrogen content was significantly enhanced, whereas phosphorus and potassium showed little enhancement. Thus, the SAE treatment is one of the superlative sustainable strategies for food, feed, and as excellent plant conditioner.

## Introduction

Nowadays, fertilizer improvement has become an urgent global concern because of economic needs (Agathokleous et al., 2022). There is anxiety about environmental pollution from the application of chemical fertilizers, which cause long-term, extensive adverse effects on soil fertility, environmental dysfunction, greater soil salinity and degeneration, the introduction of cadmium into the crops *via* the application of P fertilizers, and higher production costs (Pahalvi et al., 2021).

Broad bean (*Vicia faba.* L., Leguminosae) crop is among the most important legume crops because of its high nutritional value that is cultivated for its green pods. It is considered a valuable and cheap protein source instead of animal protein. Also, it increases soil fertility nitrogen fixation, and the beans, husks, and hay are used as animal fodder (Hassan and Ghani, 2001).

On the other hand, sunflower (*Helianthus annuus* L., Asteraceae) is the world’s fourth largest oil crop containing high-quality edible oil. It has excellent nutritional properties and is easily cultivated and grown in different conditions and soils. The phytoremediation properties of *Helianthus* are widely established. Also, it could be composted and returned to the soil as fertilizer (Fozia et al., 2008)

Hence, organic fertilizers are strongly recommended for more agricultural safety. The term “bio-fertilization” refers to a sustainable agriculture method that involves the use of bio-fertilizers to improve soil nutritional quality, resulting in greater yields. Macroalgal extracts have really been applied as agricultural biostimulants (Abs) to enhance plant growth and development (Ammar et al., 2022).

Seaweeds include a wide variety of antioxidants, plant growth promoters, minerals, and other unique biomolecules. In addition, stimulation of plant defense mechanisms *via* polysaccharides and oligosaccharides derived from seaweeds is a potential protective strategy (Benhamou and Rey, 2012). Importantly, marine macroalgae are renewable, biodegradable, eco-friendly, and cost-effective sources of biofertilizer (Ammar et al., 2022). Natural seaweed as a biofertilizer can substitute the synthetic fertilizer that has reduced soil fertility, turning it more acidic and unfavorable for growing crops (Pahalvi et al., 2021).

Recently, several studies reported the stimulatory effect of seaweeds extracts on different legumes and Asteraceae crop plants. A strong response of the three peanut varieties was caused by applying the *Sargassum vulgare* extract, which led to higher antioxidant activity (Ben Ghozlen et al., 2023). Seaweed organic fertilizer combined with standard NPK dosage can increase vitamin C and fiber content in bush beans (Rahayu et al., 2021). In two different chickpea genotypes, several agronomical parameters recorded maximum values in treatment with the *Ascophyllum nodosum* extract (Kurakula and Rai, 2021). On the other hand, soil application of mineral fertilizer combined with foliar spraying of seaweed extracts was most beneficial for the yield and quality parameters of globe artichoke plants (Elsharkawy et al., 2021; Petropoulos et al., 2022). In addition, a foliar fertilizer extract from seaweed achieved better yield and quality for lettuce crop (Hoa et al., 2022).

The goal of our effort is to appraise the impact of seed presoaking and irrigation by the *Sargassum polycystum* aqueous extract (SAE) on germination, plant vegetative growth and different crucial metabolic contents of two socioeconomic crops in Egypt, *Vicia faba* and *Helianthus annuus*. We tried to clarify the vital role of the SAE secondary metabolites that greatly affect the nutritional quality of the two tested crops. Moreover, we shed the light of the co-benefits of using the algal extracts of brown seaweed; as a biostimulant in different commercially important applications such as human consumption, animal nutrition, and improving the soil health for facing the incident climatic changes and the food crises that strike our planet nowadays.

## Materials and methods

### Seaweeds collection and preparation

The samples of the seaweed, *Sargassum polycystum*, were taken in July 2020 from the coast-line region near the industrial area in Jizan city, which is located on the Red Sea in the Kingdom of Saudi Arabia (about 16°49’20.8” North, 42°37’17.0” East). On-site seaweeds were rinsed with seawater, tap water three times, and bottled drinking water and morphologically recognized on genus level (Guiry, 2010). Shade-dried seaweeds were 40°C for 7 days. Dry algal biomass was chopped, processed, and sieved using 2 mm screen for future use.

### Algal extract preparation

In a 250 ml flask, 5 g of dried and grinded algal biomass were added to 100 ml of distilled water. The mixture was kept in a water bath at 40 ° for 12 h, then filtered using Whatman number 1 filter paper to get a pure seaweed aqueous extract. After that, the prepared extract was preserved at 4° for further work.

### Plant materials

Healthy-looking and uniform-sized seeds of *Vicia faba L*., Leguminosae and *Helianthus annuus* L., Asteraceae were obtained from the Agriculture Research Centre (ARC), Giza, Egypt.

### Bioassay for the effect of algal aqueous extract

Twenty seeds from each of the two tested plants were surface sterilized using 0.01% HgCl_2_ for one minute and rinsed gently with sterilized distilled water. Twenty seeds from each tested plant were presoaked in the SAE. Simultaneously, another 20 seeds of each crop were treated with distilled water only as control samples, then kept on filter paper (Whatman No. 1) inside sterilized Petri dishes (9 cm) at room temperature (28°С ± 1). For both control and treatment seeds, each filter paper was kept moist *via* regular tap water addition.

### Germination parameters

Germination percentage (GP), the seedling Vigor index (SVI), as well as seedling growth characteristics, such as radicle length (RL; cm), plumule length (PL; cm), seedling height (SH; cm), seedling fresh weight (FW; g seedlings^−1^), and seedling dry weight (DW; g seedlings^−1^), were also determined 10 days after sowing (10 DAS).

Germination (%): the number of normal seedlings was counted according to the following formula:


$$\text{Germination (\%) =}\frac{\text{No}.\text{of germinated seeds}}{\text{Total No}.\text{ of tested seeds }}x100$$

Seedling Vigor index (SVI) was calculated according to Abdul-Baki and Anderson (1973) using the next formula:

SVI = Germination (%) * Total seedling length (cm)

### Treatments pattern and experimental design

The experiment was performed with 40 plastic pots containing soil mixture (sand: clay, 1:2 V/V) and watered with water holding capacity using a completely randomized block design (CRD). Fertilizers such as superphosphate and urea were put in all pots. The 40 pots were then divided into four groups (treatments). Each group has ten pots, one for each treatment’s replication. The sterilized seeds of *Vicia faba* L. and *Helianthus annuus* L. were separately soaked in deionized water (50 seeds of each strain for control). Before sowing, the other 50 seeds of each strain were individually soaked in *Sargassum* aqueous extract (SAE). The control and treated seeds were irrigated by tap water except for irrigation practice on the 8th day after sowing, where the treated plants were irrigated by the mixture of algal extract and water (1:3 V/V), while the control plant was normally irrigated by tap water. Plants were picked 45 days after sowing (DAS) to evaluate growth biomarkers and physiological characteristics.

### Growth biomarkers

After eliminating soil particles from the roots and washing them with distilled water, the shoot length was measured on a meter scale, and the fresh biomass of the shoot was measured using a weighing balance. All samples were kept in the oven for 48 h at 70 °. After allowing the dried plants to cool at room temperature, they were weighed again to record the shoot dry weight. The plant moisture content was calculated on the basis of wet weight according to by means of the following formula:


Moisture content (%) =W2 −W3 x 100W2 − W1

W1 = weight of the empty container with lid

W2 = weight of the container with lid and sample before drying

W3 = weight of the container with lid and sample after drying.

The number of leaves per plant was counted. The leaf area (LA) per plant was determined using the squared papers method and using the equation,

M2 = M1 x W2/W1, (Haroun, 1985)

Where M1 and W1 are the area and weight of the square paper and M2 and W2 are the area and weight of the plant leaf, respectively.

Leaf area index (LAI) was calculated according to Amanuallah et al. (2007) using following formula,

Leaf Area Index = Leaf area per plant (m^2^) x No. of plants per m^2^

### Physiological measurements

#### Extraction and estimation of photosynthetic pigments

For the determination of different pigments, we applied the methods authorized by Arnon (1949) and Horvath et al. (1972) and modified by Kissimon (1999). Briefly, the plant leaves were carefully ground with 80% acetone for 5 minutes. After 3 minutes of centrifugation at 1000 rpm, the supernatant was measured at 480, 644, and 663 nm wavelengths.

#### Estimation of carbohydrates content

Soluble sugar extraction from pre-dried samples of plant shoots was performed using 80% ethanol determined according to the anthrone sulfuric acid procedure (Whistler et al., 1962). Polysaccharides concentrations were estimated in plant residue left after the extraction of soluble sugars. Finally, the total content of carbohydrates was determined by the summation of the total contents of polysaccharides and soluble sugars for each sample. All results were recorded as mg 100 g^-1^ DW of shoots.

#### Assay of total phenolics

The Folin-Ciocalteu reagent was used in accordance with Singleton and Rossi’s (1965) methodology to determine the total phenolic content of the plant. At a wavelength of 725 nm, the absorbance was measured. Total phenolics were determined using a standard curve, and the results were expressed as gallic acid equivalents (GAE) in milligrams per one hundred grams of dried material.

#### Assay of total flavonoids

The Aluminum Chloride Calorimetric Assay was used to derive an estimate of the total flavonoid content (Zhuang et al., 1992). The total flavonoid was determined using the standard plot, and the results were presented in the form of mg catechin equivalent per 100g of dried sample.

#### Assay of total antioxidant capacity

The phosphomolybdenum method measured the plant methanolic extract’s antioxidant capability (Prieto et al., 1999). Ascorbic acid equivalent mg/100g dry sample represents the antioxidant activity.

#### Quantification of total nitrogen

The total plant nitrogen was evaluated by the micro- Kjeldahl method (Pregi, 1945). Titration against a standard sulphuric acid (0.0143 N) was used to determine each sample, with bromocresol green and methyl red (3:2 v/v) as indicators until the end point (a faint red color) was reached. The titration figures were converted into mg nitrogen using:

1 ml of 0.0143 NH_2_SO_4_ = 0.28 mg Nitrogen

#### Estimation of potassium and phosphorus

Based on the wet ashing method, the dried plant matter was digested according to Chapman and Pratt (1962). Potassium was determined by the flame emission technique as adopted by Ranganna (1977). Phosphorus was estimated simultaneously by inductively coupled plasma optical emission (ICP) Spectrometry using the method of Soltanapour (1985). Data were calculated as ppm.

### Qualitative analysis of phytochemical substances in algal extracts

The *S. polycystum* extract was phytochemically screened using Harborne’s method (1998). The alkaloids, terpenoids, steroids, tannins, saponins, flavonoids, phenols, coumarins, quinones, and glycosides were identified *via* phytochemical screening. The general responses that took place throughout these studies revealed whether or not the algal extracts that were analyzed contained these chemicals.

Test for Alkaloids: Alkaloid identification required 2 mL of strong hydrochloric acid and 2 mL algal extract. Mayer’s reagent was added. Alkaloids appear green or white.

Test for Terpenoids: Terpenoids were identified by adding 2 milliliter chloroform and concentrated sulphuric acid to 0.5 ml algal extract. A reddish brown interface shows terpenoids.

Test for Steroids: 2 mL chloroform and 1 mL sulphuric acid were added to 0.5 mL algal extract to identify steroids. Steroids cause reddish brown interface rings.

Test for Tannins: The algal extract was mixed with 1 mL of 5% ferric chloride to identify tannins. Tannins cause dark blue or greenish-black.

Test for Saponins: 2 mL distilled water and 2 mL algal extract were agitated in a graduated cylinder for 15 min to identify saponins. Saponins cause 1 cm foam.

Test for Flavonoids: 2 mL algal extract was mixed with 1 mL 2N sodium hydroxide to identify flavonoids. Yellow denotes flavonoids.

Test for Phenols: Adding 2 mL of distilled water and a few drops of 10% ferric chloride to 1 mL of algal extract identified phenols. phenols cause blue/green color.

Test for Coumarins: In order to identify the coumarins, 1 milliliter of algal extract was mixed with 1 milliliter of 10% sodium hydroxide. The appearance of a yellow color is a clear signal that coumarins are present.

Test for Quinones: Algal extract was mixed with 1 mL concentrated sulphuric acid to identify Quinone. Red color denotes quinones.

Test for Glycosides: Glycosides were identified by adding 3 mL chloroform and 10% ammonium solution to 2 mL algal extract. Pink denotes glycosides.

### Statistical analysis

All experiments were conducted in three replicates, and the results are recorded as the mean ± standard deviation. For statistical purposes, one-way ANOVA was applied to test the significant differences between treatments using the statistical software SPSS (IBM, v. 25) treatments followed by *post hoc* Duncan’s test to determine the difference in growth and metabolic parameters between the target treatment group and the control group at a probability level (P) ≤ 0.05.

## Results

### Effect of *Sargassum polycystum* aqueous extract on the germination traits

**Figure 1:**
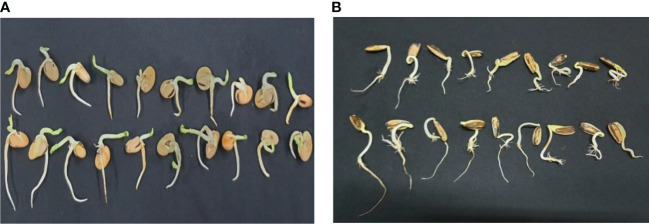
Effect of presoaking in *Sargassum polycystum* aqueous extract (SAE) on the germination of *Vicia faba* and *Helianthus annuus*, 10 days after sowing **(A)** Control and treated *Vicia* plants, **(B)** Control and treated *Helianthus* plants.

The data documented in **Table 1** and illustrated by **Figure 1** revealed that SAE does not actually affect the germination percentage as it recorded 100% for both control and treated *V. faba* and *H. annus* plants. At the same time, the seedling vigor index (SVI) improved by (24.09%) and (22.73%) in *V. faba* and *H. annuus*, respectively, over their controls.

**Table 1:** Effect of presoaking in *Sargassum polycystum* aqueous extract on germination parameters of *Vicia faba* and *Helianthus annuus* plants.

		Plumule Length (cm)	Radicle Length (cm)	Seedling total height (cm)	Seedling fresh weight (g)^-1^	Seedling dry weight (g)^-1^	Seedling Water content	Seedling vigor index
*V.faba*	Control	2.856^a^ ± 0.573	4.730^b^ ± 0.839	7.156^b^ ± 1.131	2.865^b^ ± 0.403	0.696^b^ ± 0.002	75.266^a^ ± 3.615	715.56^b^ ± 113.148
	Treated	3.720^a^ ± 0.783	5.570^a^ ± 1.462	8.880^a^ ± 2.371	3.385^a^ ± 0.497	0.752^a^ ± 0.002	77.265^a^ ± 4.011	888.00^a^ ± 237.056
*H.annuus*	Control	6.579^b^ ± 1.218	2.926^b^ ± 0.738	9.715^b^ ± 1.271	1.145^b^ ± 0.115	0.070^b^ ± 0.002	93.864^b^ ± 0.657	971.48^b^ ± 127.144
	Treated	7.935^a^ ± 1.061	4.132^a^ ± 4.132	11.923^a^ ± 2.193	1.507^a^ ± 0.218	0.075^a^ ± 0.001	94.923^a^ ± 0.587	1193.16^a^ ± 219.340

^a, b^significantly different according to DMRTs at 0.05 level.

Although the SAE treatment non-significantly enhanced the length of plumule and seedling dry weight of *Vicia* plants (30.25% and 8.05%) over the control plants, all the other estimated germination traits were significantly increased (17.76%, *Helianthus* plumule length), and (24.09% *Vicia* radicle) and (20.16% *Helianthus* radicle), (41.22% *Vicia* seedling height) and (22.73% *Helianthus* seedling height). The fresh weights were significantly induced up to 18.15% and 31.62% in *Vicia* and *Helianthus*, respectively, whereas *Helianthus* dry weight raised by 7.14% over the control plants.

### Effect of presoaking and irrigation by *Sargassum polycystum* aqueous extract on growth biomarkers

#### Shoot growth criteria

Biostimulants are natural organic compounds that, in low concentrations, can improve plant growth, nutrient absorption, stress tolerance, and crop productivity. Except for *Helianthus* dry weight, data itemized in **Table 2** showed that the SAE exhibited a significant effect on all estimated morphological traits. However, *faba* plants were more affected by the SAE, as it achieved a higher increment in shoot length, shoot fresh and dry weights, and shoot water content. These boosts were evaluated by 49, 32, 19, and 1.9%, respectively, compared to the *faba* control. The corresponding improvements for the same growth criteria were estimated by 15, 18, 33, and 1.05% one-to-one in *Helianthus* shoots.

**Table 2:** Effect of presoaking and irrigation by *Sargassum polycystum* aqueous extract on the growth biomarkers of *V. faba* and *H.annuus* plants.

		Shoot length (cm)	Shoot fresh weight (g)	Shoot dry weight (g)	Water content %	Leaf number per plant	Leaf area (mm^2^)/plant	Leaf area index	Number of root nodules	Number of Flowers
*V.faba*	Control	13.64^b^ ± 1.05	2.62^b^ ± 0.11	0.35^b^ ± 0.01	86.49^b^ ± 0.39	10.4^b^ ± 0.89	46.49^b^ ± 2.56	.0017^b^ ± 0.0	1.6^b^ ± 0.548	5.8^b^ ± 1.304
	Treated	20.32^a^ ± 1.86	3.45^a^ ± 0.53	0.41^a^ ± 0.05	88.13^a^ ± 0.63	12.4^a^ ± 0.55	55.56^a^ ± 3.95	.0020^a^ ± 0.0	2.8^a^ ± 0.837	8.6^a^ ± 1.140
*H. annuus*	Control	19.04^b^ ± 1.27	2.32^b^ ± 0.23	0.17^a^ ± 0.02	92.39^b^ ± 0.24	4.8^b^ ± 1.1	24.56^b^ ± 2.51	.0009^b^ ± 0.0	–	–
	Treated	21.82^a^ ± 0.4	2.74^a^ ± 0.14	0.18^a^ ± 0.02	93.36^a^ ± 0.44	6.4^a^ ± 0.89	26.81^a^ ± 0.99	.0010^a^ ± 0.0	–	–

^a, b^significantly different according to DMRTs at 0.05 level.

#### Leaf growth parameters

Data which were enumerated in **Table 2**, exposed that the SAE significantly increased leaves number/plant (19 and 33%), leaf area (19.51 and 9.16%), and leaf area index (17.65 and 11.11%) one-to-one in *V. faba* and *H. annuus* respectively.

#### Flowering and nodulation

**Figure 2:**
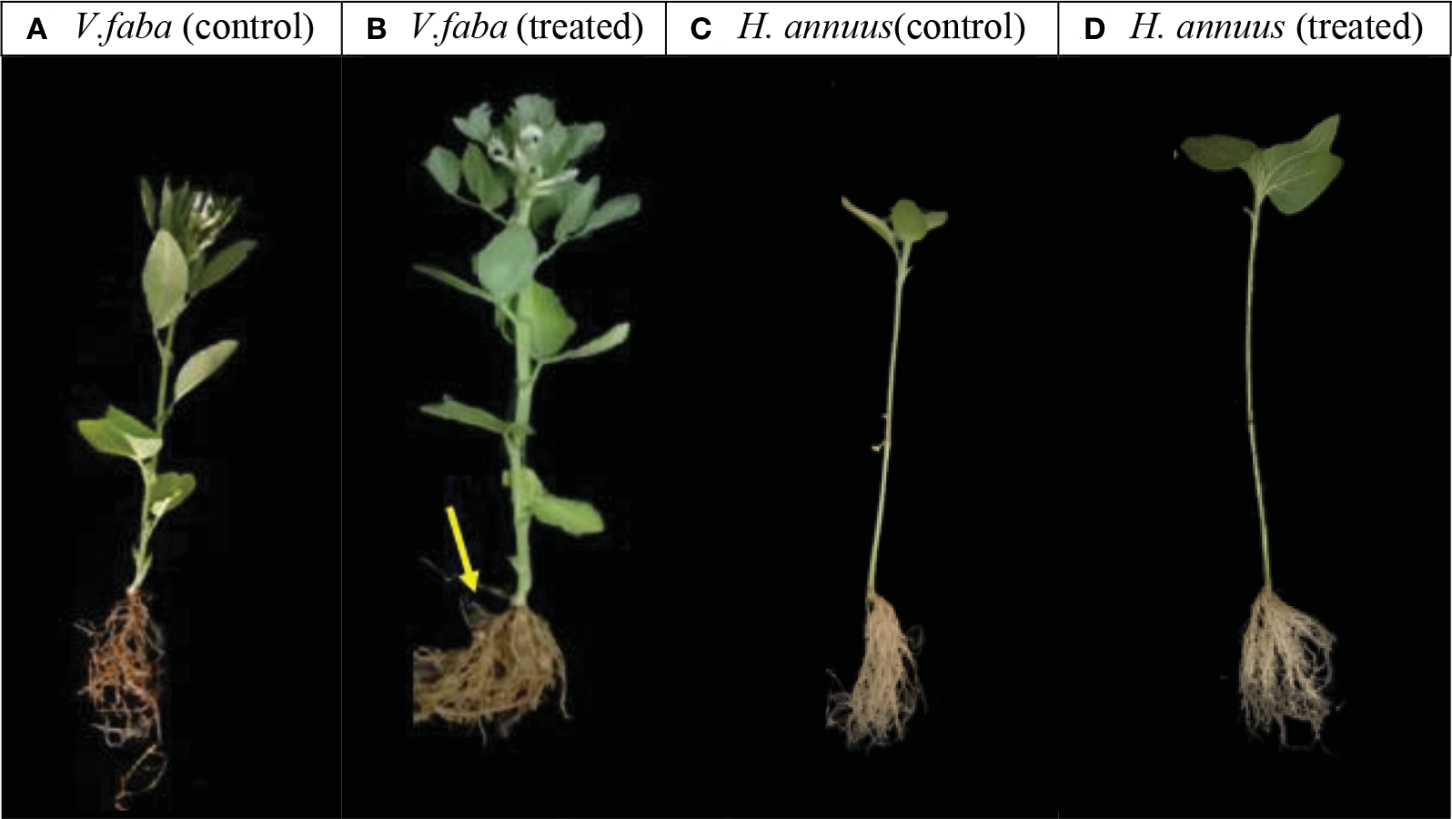
Effect of presoaking and irrigation by *Sargassum polycystum* aqueous extract on shoots and Roots morphology of treated *V. faba* **(A, B)** and *H. annuus* **(C, D)** 45 days of planting.(Yellow arrow shows nodule size in the treated *Vica* plants).

**Table 2** exposed that the algal treatment produced more than two times (55.6%) root nodules in *Vicia* roots as compared to their control and enhanced nodules size remarkably (**Figure 2**). With respect to flower formation, the employed SAE treatment significantly amplified the flower number in *V.faba* only (**Table 2**), while the *H. annuus* did not bloom till the end of the experiment schedule (45 DAS). In addition, the SAE significantly enhanced flowering in faba plants (48.3%).

### Effect of presoaking and irrigation by *Sargassum polycystum* aqueous extract on the photosynthetic pigments

**Figure 3:**
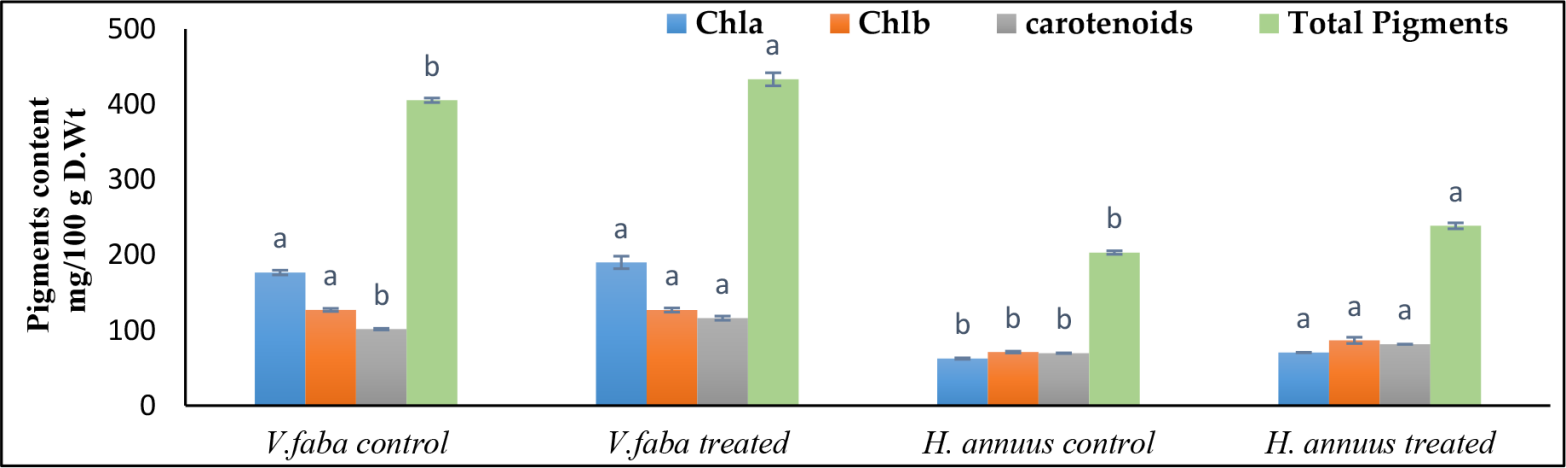
Effect of presoaking and irrigation by *Sargassum polycystum* aqueous extract (SAE) on the photosynthetic pigments of *Vicia* and *Helianthus* plants 45 days after sowing. Bars with different letters are significantly different according to DMRTs at 0.05 level.

Although, the SAE treatment significantly increased the carotenoids and total photosynthetic pigments by 14.34% and 17.09% and 7% and 17% over the control plants of *Vicia* and *Helianthus* in that order (**Figure 3**). Chlorophylls a and b were not significantly affected in *Vicia* but significantly affected in *Helianthus* plants.

### Effect of presoaking and irrigation by *Sargassum polycystum* aqueous extract on the carbohydrate content

**Figure 4:**
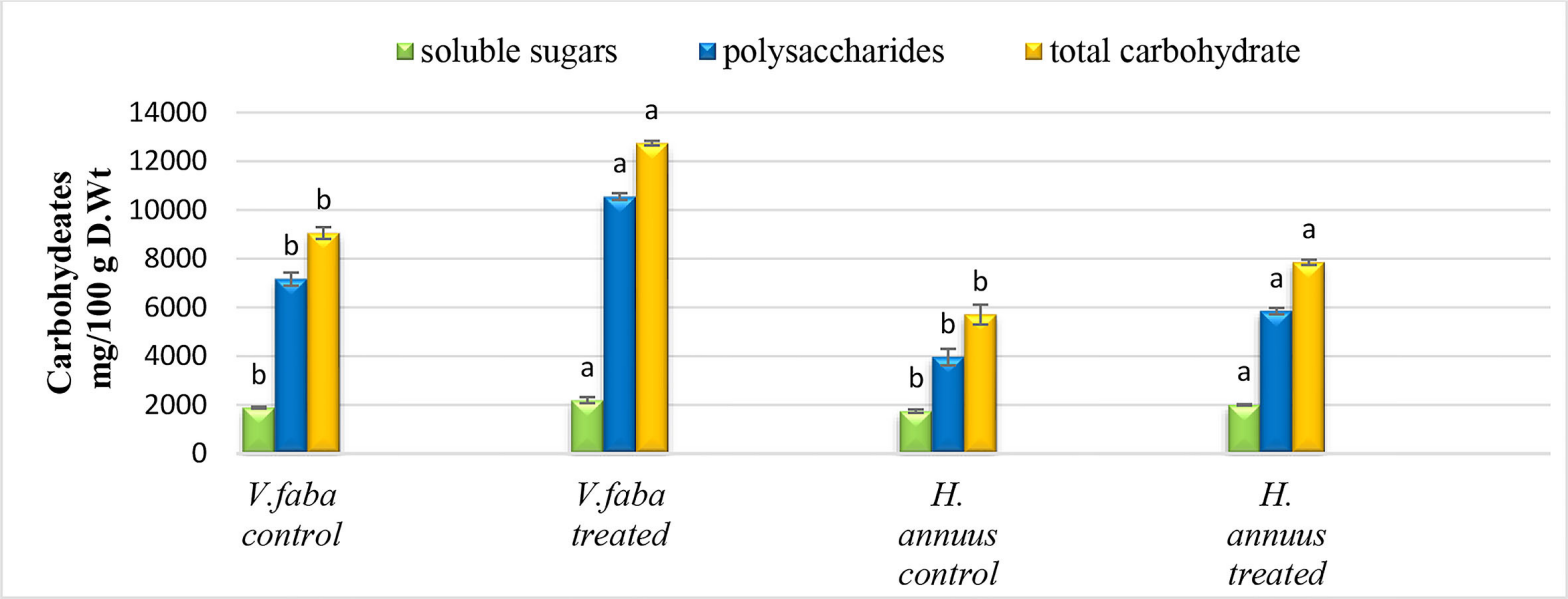
Effect of presoaking and irrigation by *Sargassum polycystum* aqueous extract (SAE) on the carbohydrates content of *Vicia* and *Helianthus* plants 45 days after sowing. Bars with different letters are significantly different according to DMRTs at 0.05 level.

Complementarily with our previous results, the SAE treatment significantly improved carbohydrate content in both crop plants. Although soluble sugars moderately enhanced (15% and 16%), polysaccharides highly improved by 47% and 48% for both plants. Consequently, total plant carbohydrate content increased by 41% and 38% for *Vicia* and *Helianthus*, respectively **Figure 4**

### Effect of presoaking and irrigation by *Sargassum polycystum* aqueous extract on some non-enzymatic antioxidants component; phenolics and flavonoids

**Figure 5:**
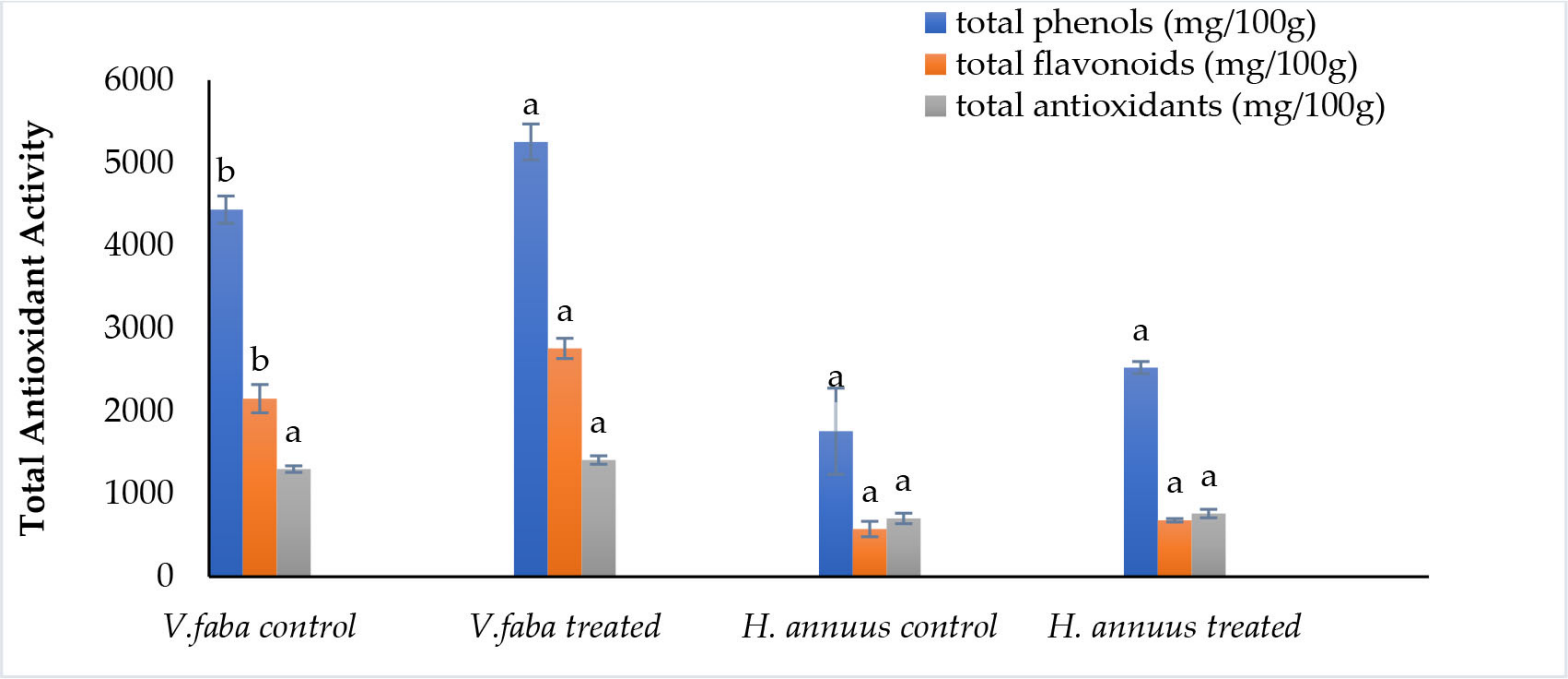
Effect of presoaking and irrigation by *Sargassum polycystum* aqueous extract (SAE) on the phenolics, flavonoids content and total antioxidant activity of *Vicia* and *Helianthus* plants 45 days after sowing. Bars with different letters are significantly different according to DMRTs at 0.05 level.

According to the data presented in **Figure 5**, phenolic and flavonoid contents were significantly induced in *Vicia* but not significantly promoted in *Helianthus* plants. Phenolic content was enhanced by 18% and 43%. Whereas flavonoids improved by 28% and 18% in *Vicia* and *Helianthus* shoots, respectively. This enrichment nominates the treated plants as preferable fodder for animal nutrition.

### Effect of presoaking and irrigation by *Sargassum polycystum* aqueous extract on the total antioxidant capacity

Referring to **Figure 5**, The total antioxidant activity was not significantly induced by 8.5% and 8.4% for *Vicia* and *Helianthus*, respectively.

### Effect of presoaking and irrigation by *Sargassum polycystum* aqueous extract on NPK content

**Figure 6:**
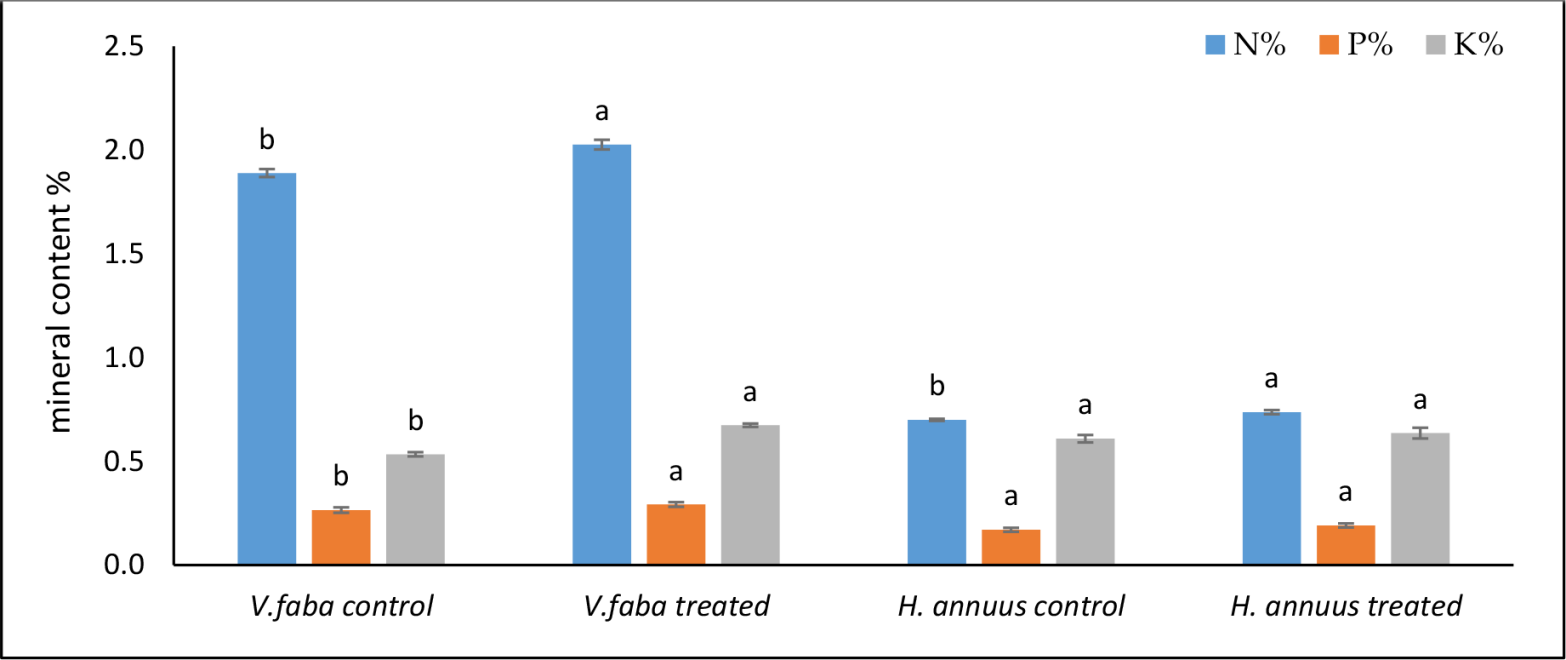
Effect of presoaking and irrigation by *Sargassum polycystum* aqueous extract (SAE) on NPK content of *Vicia* and *Helianthus* plants 45 days after sowing. Bars with different letters are significantly different according to DMRTs at 0.05 level.

The data in **Figure 6** exposed that the algal extract application significantly promoted the production of the total -N content by 7% and 5% in *Vicia* and *Helianthus* compared to untreated plants. Although The phosphorus and potassium contents were significantly stimulated by the SAE in *Vicia* plants (10% and 26%), The situation was different in *Helianthus*, where phosphorus and potassium non significantly raised (12% and 4%) in *Helianthus* shoots.

## Discussion

Among important sources of biostimulants are humic acid, chitosan, fungi, beneficial bacteria, and seaweed extracts (El-Beltagi et al., 2019). Seaweeds show an array of physiological and biochemical characteristics. Macroalgal extracts are rich sources of plants growth regulators (auxins, gibberellins, cytokinins and abscisic acid) in addition to the macronutrients, and micronutrients as well as amino acids and vitamins (Issa et al., 2019) and polysaccharide and oligosaccharide contents (Benhamou and Rey, 2012).

Our findings in **Table 1** are consistent with the results of El-Sheekh and El-Saied (2000), who documented that the crude extract of the green seaweed *Corallina mediterranea* enhanced seed germination, root and shoot length and lateral roots number of *Vicia faba*. In harmony, Baroud et al. (2019) documented that *Fucus spiralis* extract improved the germination percentage, radicle and hypocotyls length, total seedling length, dry weights and seedling biomass of pepper (*Capsicum annuum*).

Raj et al. (2015), reported the existence of steroids in the methanol extract of *Sargassum tenerrimum*. Sterols are cell membrane structural components that modulate membrane permeability and fluidity. Sterols, as a component of the algal extract, have been proven to have a beneficial influence on seed germination. For example, seeds of transgenic *Brassica juncea* overexpressing the HMG-COA SYNTHASE gene resulted in greater sterol centration and speeder germination than the wild type (Wang et al., 2012). Moreover, Vriet et al. (2012), stated that BRs and sterols are involved in germinating seeds under regular and stressed conditions. The incident growth improvement that happened in the *vicia* and *Helianthus* plumule, radical, shoot and root criteria (**Tables 1**, **2**) may be attributed to the existence of the plant growth hormone as a component of the seaweed extract as recorded by the speculation of Crouch and Van Staden (1993) when cited that, the existence of a broad spectrum of plant growth-promoters is related to the wide variety of growth responses elicited by seaweed extracts. This interpretation has strongly supported the role of auxins and cytokinins, which regulate shoot and root development (Kurepa and Smalle, 2022).

In the same connection, Baroud et al. (2019) reported that plants treated with (2%) of the Bifurcaria bifurcate and *Fucus spiralis*, aqueous extract showed higher t growth biomarkers (root and shoot length, total plant height, and dry weight). Leaf length did not differ between cultivars, but leaf area index (LAI) and leaf number may differ between them throughout the growing season (Castro-Nava et al., 2016). The leaf area index (LAI) is a non-dimensional variable used to evaluate different agronomic traits such as canopy, photosynthesis and evapotranspiration, which play a vital function in the transformation of energy and mass between the atmosphere and plant canopy in an ecosystem (Law et al., 2001).

Leaf area index (LAI) is related to liquid photosynthesis performed by crop plants (Wiedenfeld and Enciso, 2008). This association exists because the LAI is closely related to the amount of light absorbed and, as a consequence, to the photosynthetic activity conducted by plants (Scarpari and Beauclair, 2009). The recorded boosting LAI (**Table 2**) as a response to the SAE treatment is probably due to the increment in leaf area and number per plant. This finding may also contribute to a considerable relation between total photosynthetically active surface and crop productivity, as documented by Sandhu et al. (2012).

The attained enhancement in *Vicia* flower numbers (**Table 2**) a result of the SAE treatment may be explicated by the existence of the phytohormone cytokinin as a constituent of seaweed extract (Issa et al., 2019). Cytokinins play a role in different aspects of plant development and growth and physiological evidence suggests that they also are involved in floral transition. D’Aloia et al. (2011) elucidated the putative roles of cytokinins during the floral transition in Arabidopsis.

Seaweeds’ hormonal profiles are very similar to those of higher plants. Many plant hormones have been discovered, such as biologically active forms of auxins, ABA, gibberellins, and cytokinins (Issa et al., 2019). Salicylic acid, ethylene, brassinosteroids, strigolactones, and jasmonates, were also detected in macroalgal extracts (Stirk and Van Staden, 2014).

In accordance, flowering enhancement may also be interpreted by the incidence of poly- and oligosaccharides in the SAE (Benhamou and Rey, 2012). Sugars, in low concentrations, control different phases of the cell life cycle, including cell differentiation, vegetative and organ development, flower and fruit production, as well as defense reactions and maturity (Ciereszko, 2018).

The intensification in nodules number in Vicia roots (**Table 1**) may reflect the enhancement in total nitrogen production in the same treated plants (**Figure 6**). In a recent study, Darwish et al. (2022) registered an improved nodulation both in nodules numbers and fresh weight in parallel with the enhancement of root weight and length by enhancing strigolactone biosynthesis in soybean plants. Strigolactones were also detected in macroalgal extracts (Stirk and Van Staden, 2014).

In coordination with our outcomes, Abd El-Gawad et al. (2015) indicated that the algal extract positively affected nodulation in *V.faba* as it enhanced both Nodule dry weight and nitrogen content. Recently Darwish et al. (2022) registered a raised nodule number, fresh nodule weight, both root length and weight, by enhancing strigolactone biosynthesis in soybean plants.

The synchronized changes in leaf area (**Table 2**) and pigment contents (**Figure 3**) faced in our investigation are powerfully supported by the postulation of Beckett et al. (1994), who assumed that seaweed extract acts as some kind of bio-stimulant which increases yield at least in two ways. First, it might have increased the source capacity of the leaves, thus increasing the available assimilation supply by increasing leaf area and photosynthetic rates. Second, the algal extract might increase seed weight *via* enhancing the sink potential of the fruit for assimilates, thus increasing cotyledon cell number and final seed mass.

In support of the former attitude, Salah El Din et al. (2008) and Scarpari and Beauclair (2009) interrelated the variation of plant photosynthesis rate to the leaf size. They suggested that, a larger leaf size has a larger surface area with more chloroplasts, receives more sunlight, and has more stomata on the surface of the leaves, which play a vital role in gas exchange during the photosynthesis process. The enhanced leaf area and photosynthetic pigments recorded by **Table 1** and **Figure 3** routinely led to elevated sugar contents (**Figure 4**) in both tested plant strains, *Vicia* and *Helianthus*, especially the levels of the polysaccharides which exceeded too much those of the soluble sugars. The last speculation may be correlated to the fact that the seaweed extracts contain polysaccharide and oligosaccharide contents (Benhamou and Rey, 2012).

In the same concern, El-Sheekh and El-Saied (2000) reported that the crude extracts of different seaweeds increased chlorophyll content and total soluble sugars in *V. faba*. The stimulated growth of seedlings was a response to the employed algal extract but to different degrees. Likewise, Baroud et al. (2019) registered a high enhancement in the total sugar content of pepper plants as an outcome of treating the plant seeds with aqueous extracts of brown algae.

In synchronization, Abbas (2013) demonstrated that algae extract positively influences various metabolic functions such as respiration, photosynthetic activity, ion uptake, as well as leaf pigmentation. Generally, the influence of SAE on chlorophyll levels may be correlated to the probable existence of betaines (Baroud et al., 2019). On the other hand, Abdel Hadi et al. (1993) attributed the high increments in growth criteria and chlorophyll contents of a soybean plant to algalization.

This elevation of sugar content (**Figure 4**) as a consequence of SAE induces appropriate cellular reactions and affects some gene expressions and metabolic processes. Sugars serve as structural and storage compounds, respiratory substrates, and intermediate metabolites in various metabolic activities. They can also serve as signaling molecules and be transported over long distances (Ciereszko, 2018).

An urgent challenge faced the animal scientists is the augmented demand for safe animal food from natural feed additives. Animal health is impacted or even regulated by antioxidant supplementation in animal feed (Christaki et al., 2020). On the other hand, agricultural waste and by-products are rich sources of valuable compounds, such as phenols and antioxidants, which could be exploited as functional elements in animal feeds (Fontana et al., 2013).

The presence of simple phenols was reported in green seaweed. Furthermore, brown macroalgae show higher contents of phenolics than other types of seaweeds. Phenolics and flavonoid compounds which has been demonstrated to exist in the tested SAE (**Table 3**), have a fundamental role in different biological activities in plants. In addition, they are believed to be secondary ROS-scavenging systems that guard the plant against diverse environmental stresses (Fini et al., 2011).

**Table 3:** Qualitative analyses of phytochemical composition of algal aqueous extracts.

Alkaloids	Terpenoids	Steroids	Tannins	Saponins	Flavonoids	Phenols	Coumarins	Quinones	Glycosides
**+**	**+**	**+**	**+**	**++**	**+**	**++**	**-**	**-**	**+**

**++**: intensely present, **+**: Present, **-**: Absent.

As presented in **Table 3**, *S. polycystum* extract contains several chemical components that may be responsible for the stimulatory effect on both tested plants. In addition, phenols were markedly presented in the extract, which may explain the increased phenolic content in the treated plants compared to the control.

From this point of view, our outcomes illustrated in **Figure 5** revealed that the SAE treatment improved both crops’ phenolic and flavonoid content. These outcomes are not only of great significance for plant growth but also to introduce these crops by products as nontoxic animal fodder (Hassan and Ghani, 2001) and hence a human health preservation target.

Adding phenolics to animal diets may have positive impacts on animal gut health, involving anti-inflammatory and antibacterial activities, saving action on vitamin antioxidants, and oxidative stability of food originating from farm animals (Starčević et al., 2015). Thus, phenolics have a known impact on meat quality directly or indirectly.

This obtained enhancement in phenolics and flavonoids contents (**Figure 5**) may be owed to the richness of seaweed aqueous and methanolic extracts by numerous metabolites such as steroids, terpenoids, phenolics, flavonoids, carbohydrates, and xanthoproteins (Raj et al., 2015; Ammar et al., 2022).

Cheynier et al. (2013) interpreted the improvement of flavonoids content to the implementation of allelochemicals which cause various alterations in secondary metabolism, especially in the synthesis of flavonoids by increasing the activity of biosynthetic enzymes such as phenylalanine ammonia-lyase (PAL), implying a shift away from sucrose production and toward repair and repair defense processes.

Similarly, the elevation of phenolics content as a result of the SAE treatment may be attributed to increased production of growth hormones and nutritional uptake in the plant roots, as well as improved polyphenol oxidase activity, which enhances phenolic buildup and thus plants antioxidant activity (Chrysargyris et al., 2018).

Marine macroalgae are subjected to both oxygen and light, resulting in the production of free radicals and different highly oxidizing factors. Yet, lacking oxidative damage in macroalgae structural elements (i.e., polyunsaturated fatty acids) and their resistance to oxidation during storage indicate that their structure has protective antioxidant defense systems. Like vascular plants, algae have protection enzymes (peroxidase, superoxide dismutase, catalase and glutathione reductase) and antioxidative molecules such as phlorotannins, tocopherols, carotenoids, ascorbic acid, phospholipids, bromophenols, and catechins (El-Beltagi et al., 2019).

Higher contents of secondary compounds such as polyphenol, flavonoid and *β*-carotene, provide the plant with a greater antioxidant capacity. Several latest studies have proven the close relationship between plant phenols, flavonoids, carotenoid content and antioxidant activity (Sarker et al., 2022; Gorni et al., 2022).

The antioxidant activities of phlorotannins isolated from *Sargassum pallidum* and *Fucus vesiculosus* have been demonstrated. Furthermore, Sulfated polysaccharides such as fucoidan, laminarin, and alginic acid from *Turbinaria* have demonstrated antioxidant activity. Many other sulfated polysaccharides extracted from seaweeds, such as sulfated galactans, galactans, sulfated glycosaminoglycan, and porphyrin, have also shown highly radical scavenging properties (El-Beltagi et al., 2019).

Seaweed extracts serve as chelators, improving the plant’s mineral and nutrient uptake characteristics while also improving the soil’s structure and aeration, promoting root growth. Seaweed extract gained this feature as it contains mineral elements, vitamins, fatty acids, and amino acids, besides the unique composition of growth regulators, which cannot normally be found in higher plants (Issa et al., 2019).

In a similar recent study, total phenols and flavonoids content and DPPH radical scavenging activity for three peanut varieties were determined after treatment with *Sargassum vulgare* extract. The data illustrate a strong response from the three peanut varieties tested. Application of algal extract promoted the antioxidant potential, corresponding to the accumulation of total phenolics and induced the accumulation of resveratrol and its derivatives leading to the highest antioxidant activity (Ben Ghozlen et al., 2023).

Saponins are structurally complex amphiphatic glycosides of steroids and triterpenoids that are widely produced by plants and also by certain marine organisms and play a role in plant development. Besides, Confalonieri et al. (2009), reported the involvement of b-amyrin and derived saponins in the regulation of root nodulation.

In a study on fenugreek seeds (*Trigonella foenum-graecum*) it was found that the diffusible saponin substances located both in the endosperm and perisperm inhibited the production of a-galactosidase activity, which is a step needed for seed germination (Zambou et al., 1993). Another role of saponins is the induction of callose synthesis in carrot cells by a spirostanol saponin (Messiaen et al., 1995). A c-pyronyl triterpenesoid saponin termed chromosaponin 1 (CSI) was reported to stimulate root growth and regulate gravitropic response by inhibiting or stimulating the uptake of endogenous auxin in root cells (Rahman et al., 2001). Furthermore, saponin affects cell elongation by inhibiting ethylene signaling (Rahman et al., 2000).

Petropoulos et al. (2022) suggested that the positive effects of applying seaweed extract on artichoke plants may be attributed to the improved nutrient uptake due to the hormone-like and chelating properties of the applied seaweed extract.

The application of seaweeds as biofertilizers in sufficient quantities improved soil conditions and crop growth parameters in different field crops. The use of seaweed biofertilizer compensated for the deficiency of N, P, and K and other minerals necessary for plant growth (Singh et al., 2016). The addition of seaweed species; *Sargassum* sp. and *Gracilaria verrucosa;* caused chemical changes in clay and sandy soils as a soil fertility indicator, improved organic content, and lowered C/N ratio in both clay and sandy soil (Ammar et al., 2022).

In accordance, Xu and Leskovar (2015) reported an increase in *Eruca vesicaria* L. antioxidant activity caused by seaweed extract treatment. In a different study, Alhasan et al. (2021), recorded a direct correlation between antioxidant activity and phenolic content. Hence, the knowledge of the nutritional contents of diverse plant residues, including their application in plant nutrition during a crop cycle, is critical, especially in sustainable agriculture (Torma et al., 2018).

Our study found that the SAE treatment improved the nitrogen and potassium content in both plant shoots and increased plant growth and final dry biomass (**Table 2** and **Figure 6**). According to Torma et al. (2018), the quantity of nutrients (particularly nitrogen and potassium) left in the soil after the crop harvest is considerable; at least 50% of the residual plant nutrients are available for the following crop cultivated without further fertilization.

*Vicia Faba* is well known for its great prominence in improving soil fertility through nitrogen fixation by root nodules, improving soil’s natural properties (Hassan and Ghani, 2001). Nitrogen-fixing bacteria (Rhizobia) coexist with legume roots and convert atmospheric N_2_ to NH_3_ by nitrogenase enzyme in plant root nodules. Furthermore, nitrogen in residual biomass of harvested crops is leached to a smaller extent than nitrogen in inorganic fertilizers, which improves groundwater quality (Aulakh et al., 2000).

On the other hand, because *Helianthus* stems contain phosphate and potassium, they can be composted and put back into the soil as fertilizer (Fozia et al., 2008). **Figure 6** shows the positive impact of the SAE in inducing a higher level of phosphorus content in both crops. Mamun et al. (2020) strengthened our findings; they reported that, instead of total nitrogen, total phosphate is the most important regulatory element for algal growth. The phosphate function in respiration is to transfer high-energy molecules such as adenosine triphosphate (ATP). Accordingly, this accessible phosphate inspires more seed respiration during *Vicia* and *Helianthus* germination, which may justify the amplified seedling growth vigor recoded by **Table 1**.

## Conclusion

*Sargassum polycystum* could be viewed as a sustainable, eco-friendly bio-based stimulant for green agriculture, as a trial to reduce the addition of harmful chemical fertilizers and face the offensive climatic changes. The qualitative analysis of the SAE opened a pandora box of varied bioactive secondary metabolites such as saponins, phenolics, flavonoids as well as the terpenes and steroids which are suggested to reinforce the vegetative growth, primary and secondary metabolites of both *Vicia faba* and *Helianthus annuus*. In addition, seaweed extract enhanced the antioxidant activity of both tested plants. Importantly, there is also a positive indication of the ability of dual utilization of *Vicia faba* and *Helianthus annuus* residual biomass in animal nutrition, as well as extraction of phytochemicals and further conversion into value-added products and utilization as safe biostimulants.

## Data availability statement

The original contributions presented in the study are included in the article/supplementary material. Further inquiries can be directed to the corresponding authors.

## Author contributions

Conceptualization: ME-S, SM, SH, and AN. Methodology: ME-S, SM, SH, MA-H, AE, AN. Validation: ME-S, SM, SH, MA-H, AE, and AN. Formal analysis: ME-S, SM, SH, MA-H, AE, and AN. Investigation: ME-S, AE, and AN. Resources: ME-S, SM, SH, MA-H, AE, and AN. Data curation: ME-S, SM, SH, MA-H, AE, and AN. Writing—original draft preparation: SM, SH, and AN. Writing—review and editing: ME-S, SM, SH, MA-H, AE, and AN. Visualization: SM, SH, AE, and AN. Supervision: ME-S, AE, and AN. Project administration: ME-S, MA, and AE. Funding acquisition: ME-S, MA-H, and AE. All authors contributed to the article and approved the submitted version.
